# Effect and Mechanism of Thrombospondin-1 on the Angiogenesis Potential in Human Endothelial Progenitor Cells: An In Vitro Study

**DOI:** 10.1371/journal.pone.0088213

**Published:** 2014-02-05

**Authors:** Qing Qin, Juying Qian, Lei Ge, Li Shen, Jianguo Jia, Jianhao Jin, Junbo Ge

**Affiliations:** Shanghai Institute of Cardiovascular Disease, Zhongshan Hospital, Fudan University, Shanghai, China; Georgia Regents University, United States of America

## Abstract

**Objective:**

Coronary collateral circulation plays a protective role in patients with coronary artery disease (CAD). We investigated whether thrombospondin-1(TSP-1) has an inhibitory effect on angiogenesis potential in endothelial progenitor cells(EPCs) and tested whether TSP-1 are altered in plasma of patients who had chronic total occlusion (CTO) in at least one coronary artery and with different collateral stages(according to Rentrop grading system).

**Methods and Results:**

We isolated early and late EPCs from human cord blood and investigated a dose-dependent effect of TSP-1 on their angiogenesis potential by Matrigel angiogenesis assay. We found that TSP-1 (5 µg/ml) inhibited early EPCs incorporation into tubules after pretreatment for 1, 6 and 12 hours, respectively (83.3±11.9 versus 50.0±10.1 per field for 1 hour,161.7±12.6 versus 124.0±14.4 for 6 hours, 118.3±12.6 versus 68.0±20.1 for 12 hours, p<0.05). TSP-1 also inhibited late EPCs tubule formation at 1 µg/ml (6653.4±422.0 µm/HPFversus 5552.8±136.0 µm/HPF, p<0.05), and the inhibition was further enhanced at 5 µg/ml (6653.4±422.0 µm/HPF versus 2118.6±915.0 µm/HPF p<0.01). To explore the mechanism involved, a small interfering RNA was used. In vitro, CD47 siRNA significantly attenuated TSP-1's inhibition of angiogenesis on late EPCs and similar results were obtained after functional blocking by anti-CD47 antibody. Then we investigated pathways downstream of CD47 and found TSP-1 regulated VEGF-induced VEGFR2 phosphorylation via CD47. Furthermore, we examined plasma TSP-1 levels in patients with CTO who developed different stages of collaterals and found a paradoxical higher level of TSP-1 in patients with good collaterals compared with bad ones (612.9±554.0 ng/ml versus 224.4±132.4 ng/ml, p<0.05).

**Conclusion:**

TSP-1 inhibited angiogenesis potential of early and late EPCs in vitro. This inhibition may be regulated by TSP-1's interaction with CD47, resulting in down regulation of VEGFR2 phosphorylation. In patients with CTO, there may be a self-adjustment mechanism in bad collaterals which is shown as low level of angiogenesis inhibitor TSP-1, and thus favoring collateral formation.

## Introduction

Coronary collateral circulation plays a protective role in patients with coronary artery disease(CAD) for they provide an alternative source of blood supply to myocardium that has been jeopardized by occlusive coronary artery disease, and thus help to preserve myocardial function[1]. In adults, collaterals are formed by microvascular blood vessel formation like capillary growth which occurs through both angiogenesis–the sprouting and proliferation of endothelial cells located in pre-existing blood vessels and vasculogenesis–de novo vessel growth or macrovascular vessels development through arteriogenesis–the transformation of small arterioles into larger conductance arteries. Endothelial progenitor cells (EPCs) residing in the bone marrow are mobilized in response to ischemia and promote vascularization by differentiating into tissue-specific vascular cells or by paracrine effect[2]. Cytokines can modulate neoangiogenesis potential of EPCs. Indeed, stromal cell-derived factor-1(SDF-1), vascular endothelial growth factor (VEGF), and macrophage migration inhibitory factor (MIF) have been confirmed to promote neoangiogenesis of EPCs[3], [4]. Thrombospondin-1 (TSP-1) has been shown to have a negative effect on endothelial colony forming cells (ECFC) angiogenic potential in patients with peripheral arterial disease. However, the role of TSP-1 in angiogenesis of EPCs in patients with CAD is still unknown.

TSP-1, a 142000-Da glycoprotein initially isolated from human platelets[5], was the first endogenous protein inhibitor of angiogenesis identified in 1990[6]. In addition to release by platelets, TSP-1 can also be secreted by macrophages, monocytes, fibroblasts, vascular smooth muscle cells (SMC), tumor cells, and endothelial cells (EC) as extracellular matrix(ECM) and then influence the function of these cells[7]. As a result, TSP-1 is involved in a wide range of physiological processes including thrombus formation, angiogenesis, tumor biology, wound healing, and vascular remodeling[7]. The study of TSP-1 as an endogenous angiogenesis inhibitor has accumulated most evidence in the past few years in the treatment of tumor. For instance, ABT510 and ABT898, which are synthetic analogs of TSP-1, have been put into clinical trials and proven useful in different types of tumors[8]–[10]. In cardiovascular disease, the function of TSP-1 in regulating angiogenesis is still not clear.

TSP-1's effect on angiogenesis occurs by modulating EC proliferation, adhesion, migration, apoptosis and by antagonizing the activity of VEGF. TSP-1 has no direct structural role in the ECM, but plays an important role in regulating tissue remodeling through binding and interacting with many cellular receptors and other ECM molecules through their various domains[11], [12]. TSP-1 is composed of a N-terminal domain, an oligomerization domain, a von Willebrand Factor type C (VWC) domain, three thrombospondin repeats (TSRs), and a signature domain comprising three epidermal growth factor (EGF)-like repeats, a calcium-binding wire and a lectin-like C-terminal globe [13], [14]. TSP-1 antagonizes VEGF via inhibition of VEGF release from the ECM and inhibition of VEGF signal transduction[15]. TSP-1 can also inhibit EC migration by binding to CD36, β1 integrin through its TSRs[16], [17]. Besides, TSP-1 can regulate angiogenesis through CD36- and CD47-dependent inhibitory effects on nitric oxide(NO)[14], [18]. However, the downstream mechanism mediating the effect of TSP-1 on EPCs is not clear.

In this study, we investigated the effect of TSP-1 on angiogenesis in EPCs, and further explored the downstream mechanism involved. We also studied the relationship between collateral formation and plasma TSP-1 levels in patients with chronic total occlusion (CTO).

## Materials and Methods

### Reagents

Recombinant human thrombospondin-1 was purchased from R&D. Recombinant human VEGF_165_ was obtained from Peprotech. Fibronectin(FN) was obtained from BD Bioscience.

### EPCs isolation and identification

#### Isolation of CD34+ cells

For studies involving human tissues we obtained ethical approval from the Human Research Ethics Committee of Zhongshan Hospital, Fudan University. The approval Project NO. 2010-109 refers to isolation of EPCs from umbilical cord blood for investigating the impact of TSP-1 on angiogenesis of EPCs. All samples were taken after written-informed consent using guidelines approved by the Human Research Ethics Committee of Zhongshan Hospital, Fudan University on the Use of Human Subjects. Mononuclear cells (MNCs) were isolated from human umbilical cord blood (HUCB) obtained from healthy donors. HUCB was collected in 50 ml Falcon tubes (BD Bioscience) containing 15 ml of the anticoagulant citrate phosphate dextrose. After collection, HUCB was diluted 1∶4 in 6% hydroxyethyl starch and after sedimentation for 1.5 hrs, supernatants were collected. After centrifugation at 1500 rpm for 15 min, supernatants were discarded and cells were resuspended in 7 ml PBS. MNCs were isolated by density gradient centrifugation, where 7 ml of cell suspension was layered onto 7 ml Ficoll (Ficoll-Histopaque 1077, Sigma) and centrifuged for 25 min at 2000 rpm. Thereafter, the interphase containing MNCs was collected, followed by two washing steps, 10 min at 1500 rpm each. The washed MNCs were then subjected to anti-CD34 microbeads (Miltenyi Biotec) using a magnetic cell sorter device (Miltenyi Biotec). Purity of the positively selected CD34+ cells, the hematopoietic progenitor cells (HPC), was assessed by fluorescence-activated cell sorting (FACS) analysis.

#### Expansion of CD34+ HPCs

The cord blood derived CD34+ HPCs were plated on fibronectin-coated tissue culture flasks and cultured in endothelial basal medium (EBM-2, Lonza) supplemented with EGM-2-MV-SingleQuots (Lonza) and 1% penicillin-streptomycin (Sigma-Aldrich) in a humidified incubator at 37°C with 5% CO2. After 4 days of culture, nonadherent cells were discarded by washing with PBS. Medium were changed every 2 days thereafter. To confirm the EPC phenotype after culturing for 7 days, adherent cells were incubated with DiI-labeled Ac-LDL (Molecular Probes) for one hour before visualizing with an inverted fluorescent microscope (Leica DM IRE2). Immunostaining with antibodies against VEGFR2(Flk-1,1∶50 dilution; Santa Cruz Biotechnology), CD31(1∶100 dilution; Cell Signaling) followed by Cy3-conjugated goat anti-mouse antibody(1∶250 dilution; Invitrogen) was performed, and then cells were visualized with an inverted fluorescent microscope (Leica DM IRE2). After culturing for 14 days, cells were stained with anti-von Willebrand factor antibody FITC (1∶100 dilution; Abcam), or stained with antibodies against VEGFR2 (Flk-1,1∶50 dilution; Santa Cruz Biotechnology), CD31(1∶100 dilution; Cell Signaling) followed by Cy3-conjugated goat anti-mouse antibody(1∶250 dilution; Invitrogen), and then visualized with an inverted fluorescent microscope(Leica DM IRE2).

### EPCs Matrigel angiogenesis

#### Early EPC incorporation into tube-like structure

Growth factor reduced Matrigel Matrix (BD Biosciences) was thawed and placed in 96-well plates at 37°C to allow solidification. After pretreatment with TSP-1 at 0, 0.1, 1, 5 µg/ml for 1, 6, 12 hrs respectively, early EPCs(2×10^3^/well) were pre-labeled with DiI-Ac-LDL, mixed with unlabeled HUVECs(1×10^4^/well), and cultured at 37°C for 8 hrs with VEGF(50 ng/ml). EPCs and HUVECs were analyzed by an inverted fluorescent microscope (Leica DM IRE2). The number of incorporated EPCs in tubules was determined in 5 random fields. The incorporation ratio of EPCs into the tube structure was calculated by the number of EPCs/field.

#### Late EPC Matrigel angiogenesis

Late EPCs (1×10^4^/well) were seeded on Matrigel as previously described and treated with TSP-1 at 0, 0.1, 1, 5 µg/ml for 8 hrs. EPCs tube formation was visualized with or without Calcein-AM(Invitrogen) staining by an inverted microscope (Leica DM IRE2) and the total length of such tube like structures was measured by Leica Qwin V3.0 software.

### Immunoblotting

After being washed three times with cold PBS, cells were lysed by scraping into RIPA buffer (Thermo Scientific) containing 25 mM Tris•HCl pH 7.6, 150 mM NaCl, 1% NP-40, 1% sodium deoxycholate, 0.1% SDS and proteinase inhibitor cocktail containing 2 mM PMSF,20 µg/ml aprotinin,10 µg/ml leupeptin. Phosphatase inhibitor cocktails (Sigma–Aldrich) was additionally added if needed. After 1 hr extraction at 4°C with rocking, insoluble material was removed by centrifugation. After boiling, lysates were resolved by SDS-PAGE, and transferred to nitrocellulose (BioRad). Blots were blocked with 5% nonfat milk in PBS with 0.1% Tween 20 (PBST). Blots were developed with diluted antibodies for CD47 (1∶500 dilution; Abcam), GAPDH(1∶2000 dilution; Senta Cruz Biotechnology), VEGFR2(Flk-1, 1∶500 dilution; Santa Cruz Biotechnology) Phospho-VEGFR2 (Tyr1175)(1∶1000 dilutions; Cell Signaling), at 4°C overnight, followed by incubation with goat anti-rabbit IgG (H+L)(DyLight 680 conjugated, Thermo Scientific) or goat anti-mouse IgG (H+L)(DyLight 800 conjugated, Thermo Scientific) for 1 hr. Blots were visualized on an Odyssey Imaging System (Licor). The intensity of the bands was quantified using the Odyssey software.

### Small Interfering RNA

One pair of siRNA oligonucleotides for human CD47 (5′-GACUUCUACAGGGAUAUUAdTdT-3′,and5′-UAAUAUCCCUGUAGAAGUCdTdT-3′), human integrin β1(5′- CUGUUCUUUGGAUAUAGUdTdT-3′, and 5′-ACUAGUAUCCAAAGAACAGdTd-3′) and a negative control siRNA (MISSION siRNA Universal Negative Control; Sigma–Aldrich) was used. HUVECs were transfected with Lipofectamine RNAiMAX transfecting reagent (Invitrogen) with target-specific siRNA (20 nmol/L) and control siRNA (20 nmol/L) in serum-free medium according to the recommendations of the manufacturer. Approximately 12 hrs post-transfection, fresh endothelial cell complete medium was added, and the cells were cultured for an additional 72 hrs for the detection of the expression of genes and proteins.

### Flow cytometry

CD34+ HPCs after magnetic bead sorting were measured by fluorescence-activated cell analysis. In brief, 100 µl of cell suspension was incubated with a human phycoerythrin(PE)-conjugated CD34 antibody (Miltenyi Biotec). FITC-labeled anti-human CD45 antibody was used for differential gating during flow analysis. FITC-labeled IgG1a (BD Bioscience) and PE-labeled IgG2b (BD Bioscience) served as the isotypic controls. Analysis was performed with an automated fluorescence-activated cell counter (Elite; Beckman Coulter) in which 10000 events were counted.

### Study population for plasma concentrations of TSP-1

Written informed consent was obtained from all participants or their legal representative for use of their venous blood for measuring of plasma TSP-1. Ethics approval was granted by the Human Research Ethics Committee of Zhongshan Hospital, Fudan University (Project NO. B2013-014). CAD patients (n = 113) scheduled for coronary angiography who had at least one total occlusion in one major coronary vessel with estimated occlusion of more than 3 months were enrolled in the study from January 2013 to July 2013. Twenty-two patients with coronary atherosclerosis but without significant coronary stenosis at coronary angiography were selected as controls. The baseline clinical and angiographic characteristics were recorded by trained cardiologists at the time of enrolment. Two independent observers, blinded to patient characteristics graded the severity of coronary artery disease and evaluated the collateral flow. Collateral filling of the recipient artery was assessed according to the Rentrop classification. Briefly, Rentrop grade is categorized as follows: 0—no filling of any collateral vessels; 1—filling of side branches of the epicardial segment; 2—partial filling of the epicardial artery by collateral vessels; and 3—complete filling of the epicardial artery by collateral vessels[19]. Collateral circulation was further classified into three groups: group I included Rentrop grade 0 and 1; group II included Rentrop grade 2 and group III included Rentrop grade 3.

### Blood collection

Blood samples were collected in BD Vacutainer plastic K2EDTA tubes (BD Bioscience). Plasma was obtained by centrifugation at 1000×g for 15 min within 30 minutes of collection. After an additional centrifugation step of the plasma at 10000×g for 10 minutes at 2–8°C, samples were immediately stored at −80°C until analysis.

### ELISA Assays

We used commercially available solid-phase ELISA methods for TSP-1 and employed the assay protocol developed by the manufacturer (R&D). The plates were analysed using the microplate reader Victor 2 Multilabel Counter (Wallac, Turku, Finland) at wavelength 450 nm. Frozen serum samples were thawed at room temperature and diluted at 1∶50(10 µL of sample+490 µL of Calibrator Diluent). Standard curve was constructed by plotting the mean absorbance at 450 nm for each standard on the y-axis against the concentration on the x-axis and draw a best fit curve through the points on the graph. Concentrations were reported as ng/mL.

### Statistical analysis

Data were expressed as mean ± standard deviation for continuous variables and proportions for categorical variables. Data were compared between two groups using independent samples t-test. Statistical comparisons among 4 groups were performed by using ANOVA-LSD or x^2^ testing when appropriate. All statistical analyses were performed using the statistical package SPSS for Windows (Version 15.0, SPSS, Chicago, USA). A P value <0.05 was considered as statistically significant.

## Results

### Purification and phenotypical characterization of HUCB-derived EPCs

**Figure 1:**
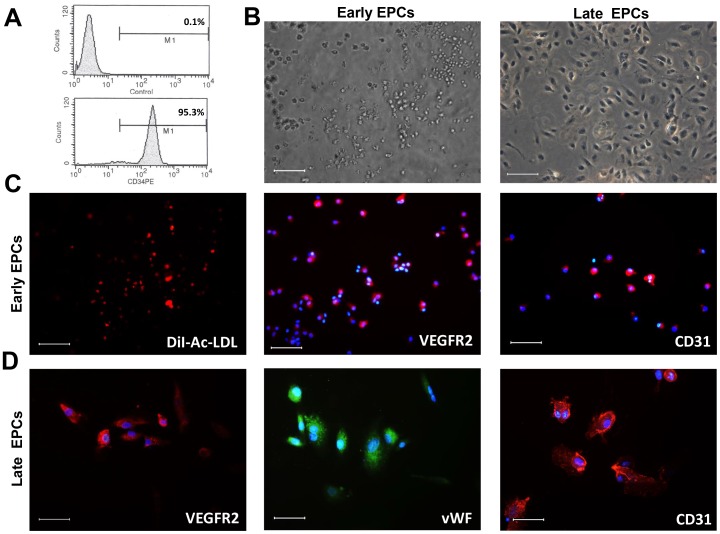
Flow cytometric analysis of CD34+ cells and morphological and immunophenotypical characterization of early and late EPCs. (A) Flow cytometric analysis of CD34 expression after isolation by anti-CD34 microbeads. Shown are representative data from 3 independent experiments using cells isolated from different cord blood with similar results. Isotype controls are used. (B) Early EPCs cultured for 7 days and late EPCs cultured for 14 days (Scale bar = 100 µm, 200×magnification). (C)Early EPCs are shown to uptake DiI-Ac-LDL(red) (Scale bar = 100 µm, 200×magnification). Immunocytochemistry of VEGFR2(red),CD31 (red), and DAPI(blue) was demonstrated in early EPCs (Scale bar = 50 µm,400×magnification). (D) Immunocytochemistry of VEGFR2(red), vWF(green), CD31(red),and DAPI(blue) was demonstrated in late EPCs(Scale bar = 50 µm,400×magnification). Shown are representative data from 3 independent experiments using early EPCs isolated from different cord blood and 3 independent experiments using late EPCs isolated from different cord blood.

Cord blood CD34+ cells were isolated and cultivated under endothelial conditions as described [20]. The purity of CD34+ cells after isolation was about 95% when analyzed by FACS (Figure 1A). After 7 days in culture, adherent cells found to endocytose DiI-Ac-LDL and immunostained positive for VEGFR2 and CD31 are early EPCs (Figure 1C) as reported [21], [22]. After 14 days of cultivation, EPCs exhibited a cobblestone morphology, spindle-like shape (Figure 1B) and displayed endothelial cell markers including VEGFR2, vWF and CD31(Figure 1D). Thus, these cells were late EPCs as reported by Hur et al [21], [22]

### TSP-1 inhibits early EPCs incorporation into vascular structure

**Figure 2:**
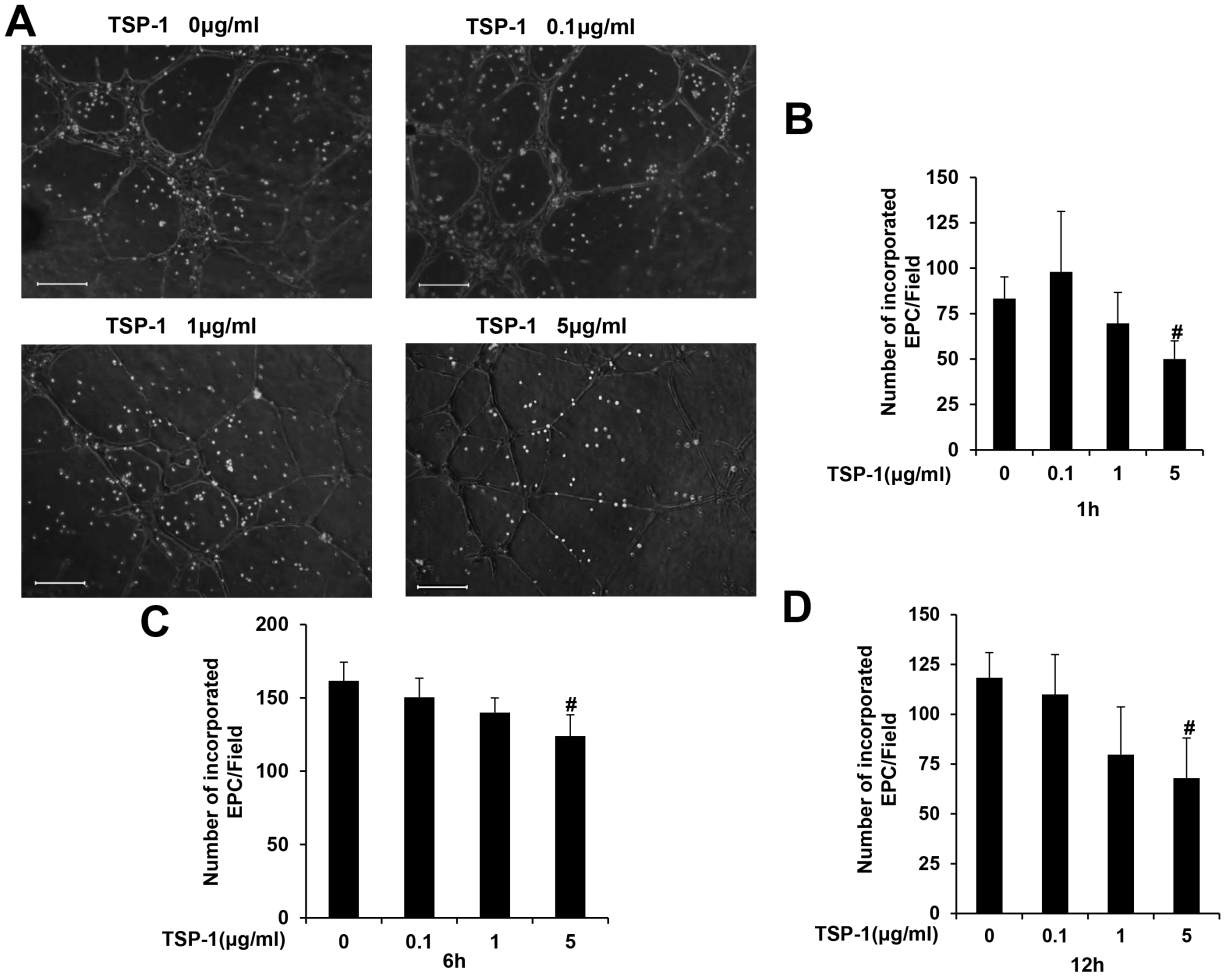
TSP-1 inhibited early EPCs incorporation into tube-like structure. (A) Early EPCs pretreated with TSP-1 at different concentrations for 1 hr were labeled with a DiI fluorescent marker(red) and coplated with HUVECs(transparent) to form tubule structures on Matrigel(Scale bar = 200 µm,100×magnification). (B–D) Early EPCs pretreated with TSP-1 at different concentrations for 1 hr(B), 6 hrs(C), 12 hrs(D) were coplated with HUVECs on Matrigel. Quantifications of incorporated EPCs per field were presented as mean±S.D of three independent experiments (#p<0.05 versus no TSP-1 intervention).

Early EPCs did not form tube-like structures on Matrigel, however, they can integrate and incorporate into vascular structure when co-cultured with HUVECs on Matrigel[21]. In order to investigate the influence of TSP-1 on vasculogenic potential of early EPCs, we co-cultured early EPCs with HUVECs after pretreatment with TSP-1 at different concentrations and different time point. Fluorescent labeling of early EPCs with DiI enabled distinction from HUVECs and analysis under fluorescence revealed that TSP-1 (5 µg/ml) inhibited early EPCs incorporation into tubules after pretreatment for 1 hr (83.3±11.9 versus 50.0±10.1 per field, p<0.05)(Figure 2A and 2B). Pretreatment of early EPCs with TSP-1(5 µg/ml) for 6 and 12 hrs, respectively, also inhibited EPCs incorporation into vascular structure (161.7±12.6 versus 124.0±14.4 for 6 hrs, 118.3±12.6 versus 68.0±20.1 for 12 hrs, p<0.05)(Figure 2C and 2D).

### TSP-1 inhibits late EPCs tubule formation

**Figure 3:**
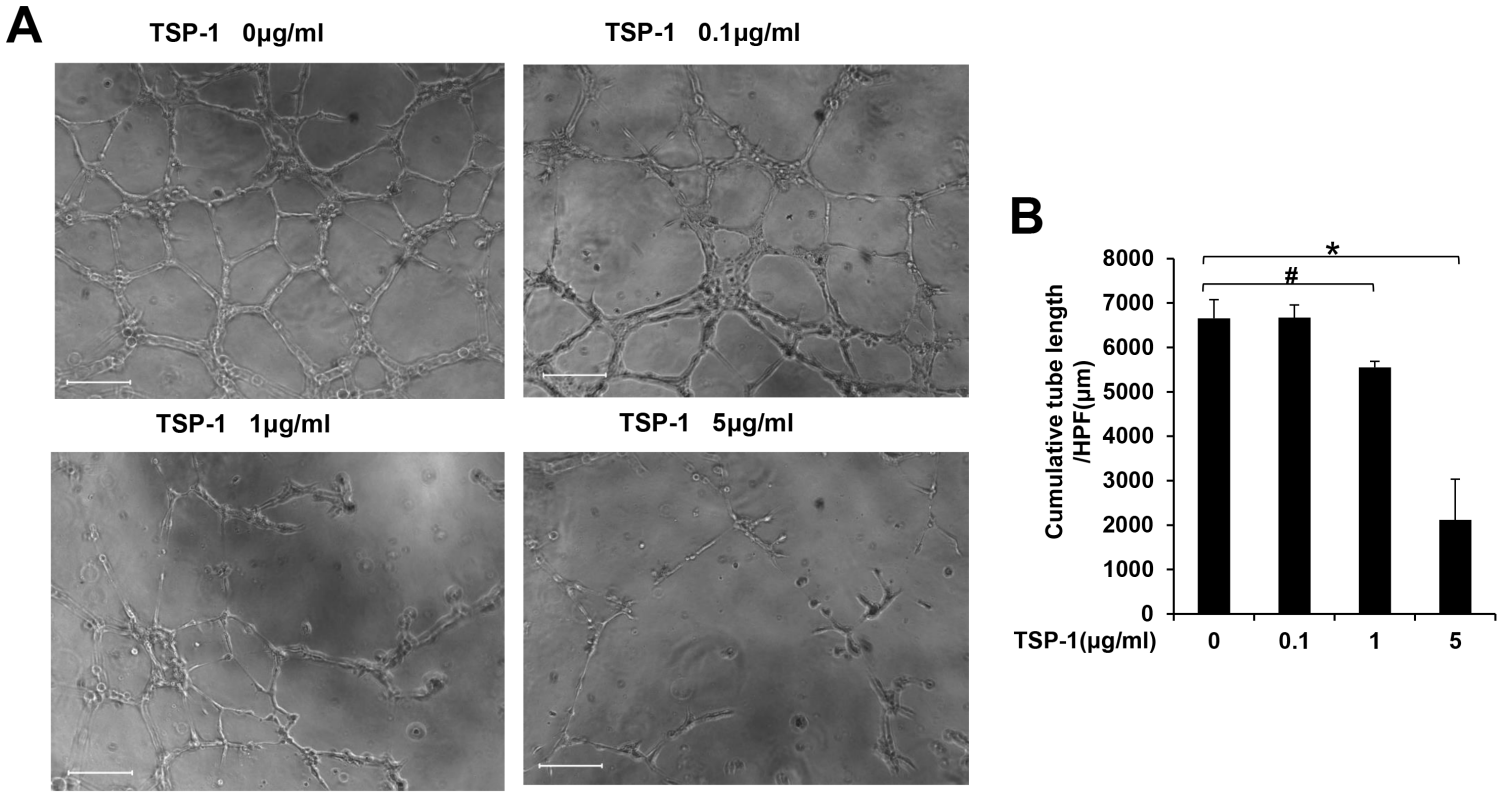
TSP-1 inhibited late EPCs tubule formation on Matrigel. (A) Late EPCs were plated on Matrigel with TSP-1 at different concentrations for 8 hrs (Scale bar = 200 µm, 100×magnification). (B) Quantification of total tube length per high power field (HPF, 100×magnification) was presented as mean±S.D. of three independent experiments (#p<0.05 versus no TSP-1 intervention, *p<0.01 versus no TSP-1 intervention).

Late EPCs exhibit capillary formation on Matrigel, therefore, we treated late EPCs with TSP-1 at 0, 0.1, 1, 5 µg/ml after EPCs were plated on Matrigel. TSP-1 inhibited late EPCs tubule formation at 1 µg/ml (6653.4±422.0 µm/HPF versus 5552.8±136.0 µm/HPF, p<0.05), and the inhibition was further enhanced at 5 µg/ml (6653.4±422.0 µm/HPF versus 2118.6±915.0 µm/HPF, p<0.01)(Figure3A and 3B). These data indicated that TSP-1 inhibits late EPCs tubule formation in a dose dependent manner.

### TSP-1 inhibits late EPCs tubule formation through CD47

**Figure 4:**
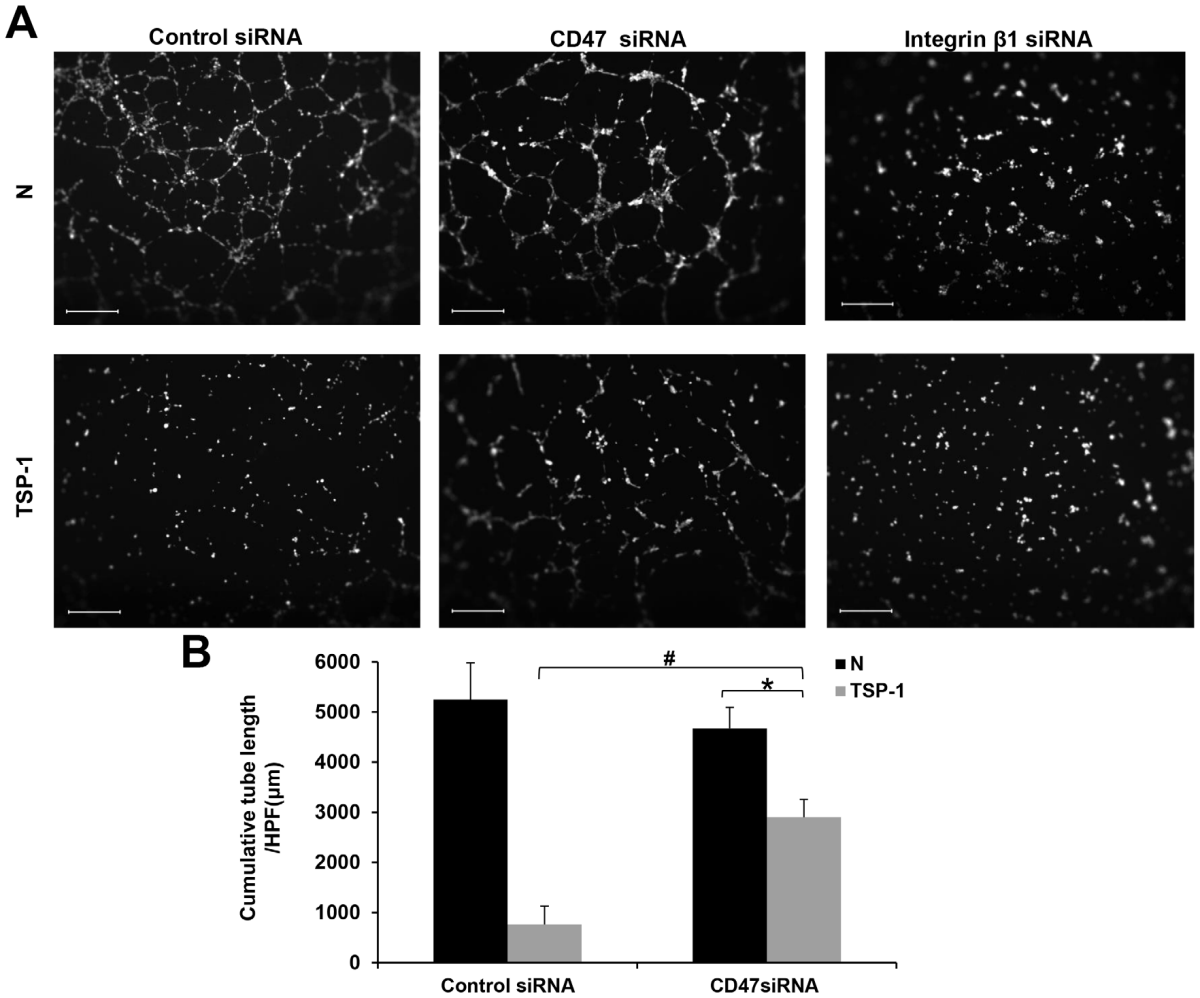
Silencing of CD47 attenuated TSP-1′s inhibition on angiogenesis of late EPCs. (A) Late EPCs were transfected with negative control siRNA, CD47 siRNA or integrin β1 siRNA, and then plated on Matrigel in the presence or absence of TSP-1(2 µg/ml) as described in methods. EPCs images were captured and analyzed by Leica Qwin system (Scale bar = 400 µm, 50×magnification). (B)Total tube length per HPF (100×magnification) was measured. Values are presented as the mean±S.D. of three independent experiments (#p<0.01 versus control siRNA with TSP-1 stimulation,* p<0.01 versus CD47 siRNA without intervention).

**Figure 5:**
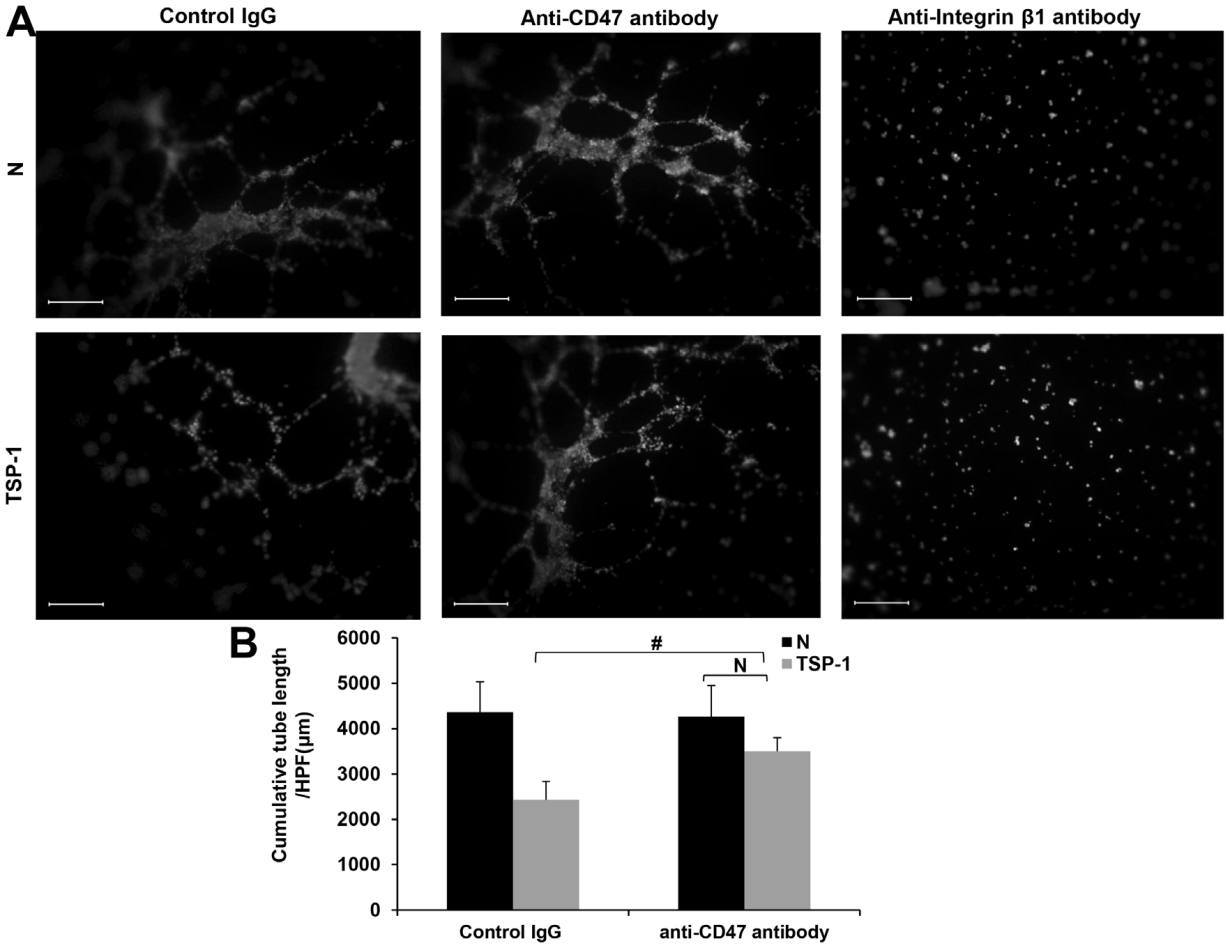
CD47 antibody attenuated TSP-1′s inhibition on angiogenesis of late EPCs. (A) Late EPCs were plated on Matrigel and treated with anti-CD47 antibody (2.5 µg/ml), anti-integrin β1 antibody (2 µg/ml) or control IgG for 30 min and then treated with TSP-1(2 µg/ml) as described (Scale bar = 400 µm, 50×magnification). (B) Total tube length per HPF (100×magnification) was measured. Values are presented as the mean±S.D. of three independent experiments (#p<0.05 versus control siRNA with TSP-1 stimulation)

The number of early EPCs increased for 2 weeks, but thereafter, they did not replicate in vitro and gradually disappeared in 4 weeks after plating. Late EPCs which appeared in 2 to 4 weeks rapidly replicated from several cells to a colony, became monolayer with almost full confluence, and showed multiple population doublings without senescence[21]. As late EPCs have more proliferation potential than early EPCs, we chose them for mechanistic study. TSP-1 can inhibit angiogenesis through direct effects on membrane receptors such as CD36, CD47 and integrins[15]. Previous study has shown that EPC-derived cells from CD34+ cord blood progenitor cells failed to express CD36[23], while they expressed a high surface density of the TSP receptor CD47[24] and β1 integrin[25]. Thus, we tested whether TSP-1 inhibits EPCs angiogenesis through CD47 or β1 integrin. Knockdown of CD47 has no effect on tubule formation of late EPCs, however, it attenuated TSP-1(2 µg/ml) induced inhibition of angiogenesis (766.7±361.7 µm/HPF versus 2903.0±356.3 µm/HPF, p<0.01). For cells with down-regulation of CD47, TSP-1(2 µg/ml) can still inhibit EPCs tubule formation (4673.0±422.3 µm/HPF versus 2903.0±356.3 µm/HPF, p<0.01)(Figure 4A and 4B). These data indicated that TSP-1 inhibited angiogenesis partly through CD47, and there may be other receptors participating in this process. Knockdown of β1 integrin inhibited EPCs angiogenesis significantly which showed its irrelevance in TSP-1′s inhibition of angiogenesis in late EPCs (Figure 4A). To confirm this, we used monoclonal CD47 blocking antibody (2.5 µg/ml) and β1 integrin blocking antibody (2 µg/ml) and obtained similar results. CD47 blocking antibody also reduces TSP-1 mediated inhibition of angiogenesis (2434±404.3 µm/HPF versus 3500.3±300.0 µm/HPF, p<0.05) while β1 integrin blocking antibody inhibited angiogenesis markedly (Figure 5A and 5B). All these data indicated that TSP-1 inhibits late EPCs angiogenesis through CD47.

### TSP-1 regulates VEGFR2 phosphorylation via CD47

**Figure 6:**
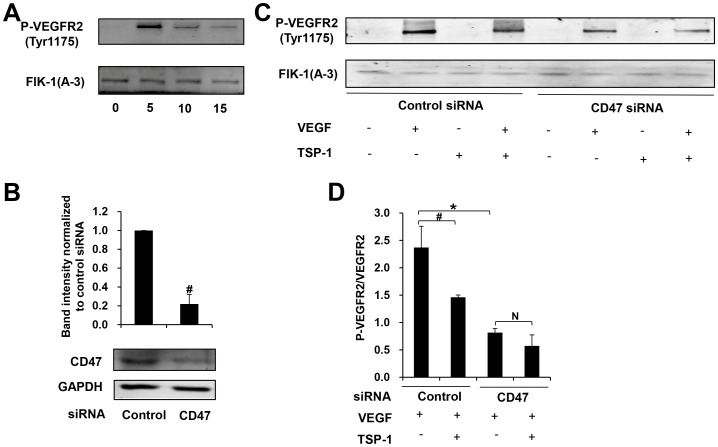
TSP-1 inhibits VEGF induced VEGFR2 phosphorylation through CD47. (A) Late EPCs were treated with VEGF (25 ng/ml) for 0, 5, 10, 15 min respectively. Total protein was extracted and the expression of FLK-1, phospho-VEGFR2 (Tyr1175) was determined by Western blot. (B)Seventy-two hrs after transfection of CD47-specific siRNA or control siRNA, the expression of CD47 in late EPCs was determined by western blotting analysis in three independent experiments (#p<0.05 versus control siRNA). (C)Seventy-two hrs after transfection of CD47-specific siRNA or control siRNA, late EPCs were treated with TSP-1(2 µg/ml) for 30 min and then VEGF(25 ng/ml) for 5 min. Total protein was extracted and the expression of FLK-1, phospho-VEGFR2 (Tyr1175) was determined by Western blot analysis. (D) Quantification of VEGFR2 phosphorylation normalized to VEGFR2 in three independent experiments(#p<0.05 versus control siRNA, *p<0.01 versus control siRNA).

In the present study, we observed that down regulation of CD47 attenuated TSP-1′s inhibition of angiogenesis. Previous study in bovine aortic endothelial cells (BAECs) and HUVECs found that CD47 associates with VEGR2 and ligation of CD47 by TSP-1 inhibits this association and VEGFR2 phosphorylation [26]. We further examined whether TSP-1 inhibited VEGFR2 phosphorylation through CD47 in EPCs. A time course using EPCs treated with 25 ng/ml VEGF demonstrated a maximal VEGR2 phosphorylation at 5 min (Figure 6A), which was used for the subsequent experiments. In our study, CD47 siRNA reached about 78% down regulation of CD47 expression in EPCs (Figure 6B). Pretreatment of TSP-1(2 µg/ml) reduced VEGF-induced VEGFR2 phosphorylation at Tyr^1175^ by about 38% (2.37±0.39 versus 1.46±0.04, p<0.05). Knockdown of CD47 inhibited VEGFR2 phosphorylation by about 65% (2.37±0.39 versus 0.82±0.08, p<0.01), whereas pretreatment of TSP-1 failed to significantly inhibit VEGFR2 phosphorylation (0.82±0.08 versus 0.57±0.2, p>0.05). Therefore, TSP-1 inhibited VEGFR2 phosphorylation through an interaction with CD47 in EPCs.

### TSP-1 in patients with chronic total occlusion

The baseline demographic and clinical characteristics of the study population are described in Table 1. Briefly, age, sex, hypertension, diabetes, dyslipidemia, smoking, previous MI, NYHA stage, eGFR, glucose, lipid profile and uric acid were consistent across all groups. Plasma concentration of TSP-1 was determined in all patients. TSP-1 levels were significantly higher in control patients (n = 22) compared with patients with CTO (n = 113) (850.0±650.6 versus 438.0±363.7, p<0.05). After categorization of collaterals according to Rentrop grading system, we found that TSP-1 levels were significantly lower in collateral group I and II as compared with control while there was no difference between collateral group III and control. Further comparison in CTO groups showed that TSP-1 was significantly higher in collateral group III compared with collateral group I (612.9±554.0 ng/ml versus 224.4±132.4 ng/ml, p<0.05) (Table 1). Therefore, these data indicate that TSP-1 has a positive correlation with Rentrop grades. Patients with CTO in collateral group I and III had worse LVEF compared with control. Analysis in CTO patients indicated that LVEF in collateral group II was better than that in collateral group I (61.5±10.0% versus 54.5±14.1%, p<0.05) (Table 1). There is also a tendency of better LVEF in collateral group III compared with collateral group I, however, no statistical significance was detected. These data showed that collateral help to rescue the function of ischemic myocardium in patients with CTO.

**Table 1:** Clinical characteristics and serum TSP-1 level in all patients.

	Control	Collateral group I	Collateral group II	Collateral group III	P
	(n = 22)	(n = 28)	(n = 48)	(n = 37)	
Age(years)	62±9	64±9	62±12	62±9	0.907
Male(%)	13(59.1)	21(75.0)	40(83.3)	32(86.5)	0.066
Hypertension(%)	15(68.2)	20(71.4)	28(58.3)	24(64.9)	0.679
Diabetes(%)	4(18.2)	10(35.7)	16(33.3)	12(32.4)	0.545
Dyslipidemia(%)	1(4.5)	2(7.1)	5(10.4)	3(8.1)	0.862
Smoking(%)	8(36.4)	15(53.6)	20(41.7)	17(45.9)	0.635
Previous MI(%)	1(4.5)	7(25)	8(16.7)	8(21.6)	0.256
NYHA stage	1.1±0.3	1.5±0.7	1.4±0.6	1.4±0.8	0.14
eGFR(ml/min/1.73m^2^)	86.8±21.9	89.6±25.6	83.4±21.2	96.0±20.4	0.08
Glucose(mmol/L)	5.7±1.5	6.4±2.3	6.4±2.3	6.0±1.7	0.432
Lipid profile					
TC(mmol/L)	4.4±1.0	4.3±1.2	4.3±1.6	4.0±1.2	0.435
TG(mmol/L)	1.8±1.0	2.1±2.2	2.2±2.0	1.8±1.7	0.677
LDL-C(mmol/L)	2.5±0.7	2.4±1.0	2.3±1.0	2.3±1.0	0.82
HDL-C(mmol/L)	1.2±0.3	1.1±0.3	1.1±0.3	1.1±0.3	0.163
Uric acid(µmol/L)	327±107	341±100	365±90	345±94	0.453
LVEF(%)	66.0±8.5	54.5±14.1*	61.5±10.0^$^	58.8±10.5^#^	0.004
TSP-1(ng/ml)	850.0±650.6	224.4±132.4*	434.7±350.2^#^	612.9±554.0^$^	0.025

LVEF indicates left ventricular ejection fraction, previous MI indicates previous myocardial infarction,eGFR indicates estimated glomerular filtration rate, TC indicates total cholesterol, TG indicates total triglyceride, LDL-C indicates low-density lipoprotein-cholesterol, HDL-C indicates high-density lipoprotein-cholesterol.

* p<0.01 compared with control, #p<0.05 compared with control,$<0.05 compared with Collateral group I.

## Discussion

TSP-1 is well characterized as an endogenous inhibitor of angiogenesis. In this study, we found that TSP-1 inhibited the angiogenesis potential of both early and late EPCs. Further study revealed that TSP-1 inhibited the tube formation of late EPCs through suppressing VEGF-induced VEGFR2 phosphorylation by interaction with CD47.

TSP-1 is a heterogeneous molecule in terms of its cellular origin and functions, different parts of the molecule showing either pro- or anti-angiogenic properties [24], [27]. According to our study in early and late EPCs, we found that intact TSP-1 protein inhibited angiogenesis potential in both cells (Figure2 and 3). The N-terminal domain of TSP-1 has a proangiogenic effect on ECFC, whereas the C-terminal region and the intact protein have antiangiogenic effects [15], [28]. Previous study showed that TSP-1 inhibits EC angiogenesis by suppressing VEGFR2 phosphorylation through interaction with CD36 and β1 integrins complex[27], [29] or CD47[26], antagonizing the proangiogenic NO signaling pathway through binding to EC surface receptors CD36[30] and CD47[31]. As EPCs had a low expression of CD36[23] but an abundant expression of CD47[24] and β1 integrin[25], we examined whether CD47 and β1 integrin were involved in TSP-1 induced inhibition on angiogenesis. Both down-regulation of CD47 expression and functional blocking of CD47 by anti-CD47 antibody showed similar results indicating that the suppression of angiogenesis by TSP-1 can be partly attenuated by inhibiting the interaction of CD47 and TSP-1 in EPCs(Figure 4 and 5). These results identified CD47 as an important receptor that mediates the anti-angiogenic function of TSP-1 in EPCs. As β1 integrin mediates the anti-migratory effects of TSP-1 in HUVECs [17], we examined the angiogenic function of this receptor in EPCs by either down regulating β1 integrin expression or blocking β1 integrin. We discovered a marked inhibition of angiogenesis by inhibiting β1 integrin activity, indicating that β1 integrin may have a pro-angiogenic property. This is consistent with the previous notion indicating that angiogenic effect of EPCs can be obtained by stimulation of β1 integrin receptor[25]. These data indicate that β1 integrin is not involved in TSP-1 induced inhibition of angiogenesis in EPCs. In our experiment, TSP-1 still exerted an inhibitory effect on EPCs after knockdown CD47 expression (Figure 4). We postulate that other receptors on EPCs may interact with TSP-1 and lead to an inhibition of angiogenesis.

CD47 is the only TSP-1 receptor that is known to mediate inhibition of VEGF signaling at physiological circulating TSP-1 concentrations. Previously, the known mechanisms through which CD47 inhibits VEGFR2 signaling include suppressing downstream NO/cGMP cascade[14] and preventing VEGFR2 phosphorylation at Tyr^1175^ which is critical for activation of downstream signaling through this receptor[26]. In our study, we studied the direct inhibition of VEGFR2 activation and found that ligation of CD47 by TSP-1 inhibited VEGFR2 phosphorylation at Tyr^1175^. We speculate that TSP-1 inhibits VEGFR2 phosphorylation by abolishing the close association of CD47 and VEGFR2, thus suppressing Akt phosphorylation and activation of eNOS, and finally resulting in attenuation of angiogenesis [26]. Based on previous data, ligation of CD47 by TSP1 also inhibits VEGF-induced cGMP synthesis [31], activation of eNOS[32], NO-stimulation of soluble guanylate cyclase[33], and activation of cGMP-dependent protein kinase[34] and this may play a role in the inhibition of angiogenesis in EPCs by TSP-1.

Abundant evidence suggests that EPCs contribute to new vessel formation after both myocardial infarction and hind-limb ischemia by differentiating into vascular cells and through a paracrine mechanism[2]. Moreover, circulating EPCs are increased in patients with good collateral grade compared to poor collateral formation among patients with CAD[35]. Together with our in vitro findings that TSP-1 inhibited angiogenesis potential of EPCs, we postulate that EPCs may have an effect on collateral formation and this may be modulated by TSP-1. As a result, we further explored the relationship between TSP-1 and collateral formation in patients with CTO. Although TSP-1 was found to be locally expressed by endothelial cells in critical leg ischemia patients and newly formed vessels [24], the change in systemic TSP-1 is still unknown in patients with totally occluded coronary arteries. In our study, plasma TSP-1 was significantly higher in good collaterals (collateral group III) compared with bad collaterals (collateral group I)(Table 1). This paradoxical increase in TSP-1 may be explained by a self-adjustment mechanism in ischemic zone. Previous data indicated that CTOs with less developed collaterals had a higher basic fibroblast growth factor(bFGF) concentration and larger gradients between collateral and coronary blood in monocyte chemotactic protein 1(MCP-1), transforming growth factor-beta(TGF-β), and placental growth factor(PIGF), which are cytokines to promote arteriogenesis[36], [37]. Collateral artery growth may have come to a halt in patients with good collaterals while arteriogenesis was preumably still ongoing in bad collaterals. As a result, a downregultion in plasma TSP-1 is reasonable to promote angiogensis in patients with bad collaterals. Platelets have been shown to release either angiogenic factors or antiangiogenic molecules, most of which are stored in α-granules during various pathological situations. Moreover, activated platelets have been shown to promote recruitment, migration, differentiation and angiogeneic potential of progenitor cells[38], [39]. TSP-1 is one of the antianiogenic molecules stored in α-granules and released during platelet activation. Indeed, angiogenic and antiangiogenic proteins have been shown to be segregated into different sets of α-granules and differential release of these α-granules may be regulated by differential G-proten-mediated signaling pathways[40]. It is likely that platelets play the regulatory role in TSP-1 levels in patients with CTO to modulte collateral formation.

### Study limitation

This is an in vitro study that partly evaluates the effect of TSP-1 on EPCs function. Furthermore, we measured systemic TSP-1 levels instead of that in collateral vessels, and this may not fully reflect the local concentration of TSP-1.

## Conclusion

TSP-1 inhibits the angiogenic potential of EPCs through CD47 mediated down-regulation of VEGFR2 phosphorylation. TSP-1 may also modulate collateral formation in patients with CTO.
